# Sex differences in brain MRI using deep learning toward fairer healthcare outcomes

**DOI:** 10.3389/fncom.2024.1452457

**Published:** 2024-11-13

**Authors:** Mahsa Dibaji, Johanna Ospel, Roberto Souza, Mariana Bento

**Affiliations:** ^1^Department of Electrical and Software Engineering, University of Calgary, Calgary, AB, Canada; ^2^Department of Radiology, University of Calgary, Cumming School of Medicine, Calgary, AB, Canada; ^3^Department of Biomedical Engineering, University of Calgary, Calgary, AB, Canada

**Keywords:** sex differences, brain MRI, deep learning, explainable AI, healthcare fairness, convolutional neural networks, neuroimaging

## Abstract

This study leverages deep learning to analyze sex differences in brain MRI data, aiming to further advance fairness in medical imaging. We employed 3D T1-weighted Magnetic Resonance images from four diverse datasets: Calgary-Campinas-359, OASIS-3, Alzheimer's Disease Neuroimaging Initiative, and Cambridge Center for Aging and Neuroscience, ensuring a balanced representation of sexes and a broad demographic scope. Our methodology focused on minimal preprocessing to preserve the integrity of brain structures, utilizing a Convolutional Neural Network model for sex classification. The model achieved an accuracy of 87% on the test set without employing total intracranial volume (TIV) adjustment techniques. We observed that while the model exhibited biases at extreme brain sizes, it performed with less bias when the TIV distributions overlapped more. Saliency maps were used to identify brain regions significant in sex differentiation, revealing that certain supratentorial and infratentorial regions were important for predictions. Furthermore, our interdisciplinary team, comprising machine learning specialists and a radiologist, ensured diverse perspectives in validating the results. The detailed investigation of sex differences in brain MRI in this study, highlighted by the sex differences map, offers valuable insights into sex-specific aspects of medical imaging and could aid in developing sex-based bias mitigation strategies, contributing to the future development of fair AI algorithms. Awareness of the brain's differences between sexes enables more equitable AI predictions, promoting fairness in healthcare outcomes. Our code and saliency maps are available at https://github.com/mahsadibaji/sex-differences-brain-dl.

## 1 Introduction

Deep learning (DL) has emerged as a powerful tool for analyzing medical image data, such as Magnetic Resonance Imaging (MRI), due to its ability to automatically extract relevant features, assisting radiologists in disease diagnosis and treatment planning (Zhou et al., 2021). However, there are some challenges in translating DL models for the clinical environment related to model reliability and explainability, limiting the usage of such tools outside research (Singh et al., 2020). The decision-making process of DL models should be understandable, allowing for validation of the clinical relevance of their findings. The results should be consistent and unbiased across various demographic groups to avoid discrimination and misdiagnosis (Chan, 2019). Consequently, models should address different sources of bias related to demographic variables like age and sex. Comprehensive model evaluations, including performance analysis on data subgroups and the application of explainable AI methods, are required (Stanley et al., 2023).

It is crucial to ensure that deep learning models perform reliably and consistently across diverse demographic subgroups, such as sex. Fairness in this context refers to the unbiased performance of DL models, where outcomes do not disproportionately favor any specific group, thus avoiding potential discrimination in clinical decision-making (Chouldechova and Roth, 2020). Studying sex differences is particularly important for understanding subtle differences that contribute to unraveling the causes of and treatments for various neurological and neuropsychiatric disorders (Mendrek, 2015). By thoughtfully incorporating sex differences into the development of DL models, we can enhance fairness, ensuring that these models deliver unbiased performance. This approach helps achieve equitable healthcare outcomes for both males and females, reducing the risk of sex-based biases in clinical decision-making.

In our previous study (Dibaji et al., 2023), we assessed the influence of sex on brain age prediction models, observing performance disparities across various sex-specific subgroups and datasets. Similarly, Piçarra and Glocker (2023) also investigated sex and race bias in brain age prediction models, highlighting the need for comprehensive bias assessment in such models. Klingenberg et al. (2023) found that MRI-based models for Alzheimer's disease detection performed significantly better for females than for males, even when trained on balanced datasets, underscoring the need for further sex-specific considerations to enhance the fairness and reliability of such algorithms. Although female and male brains are similarly structured, differences exist in the overall brain volume, as well as in cortical and subcortical regions (Williams et al., 2021). Understanding these nuances is beneficial for refining disease diagnosis and comprehending why the prevalence and prognosis of certain neurological diseases differ between males and females (Sanchis-Segura et al., 2022). Specifically, leveraging this knowledge could optimize DL models, enhancing the performance for both demographic groups.

Several studies have focused on classifying sex from brain medical images using Machine Learning (ML) methods. A common challenge addressed in these studies is the Total Intracranial Volume (TIV) difference between males and females, with TIV being, on average, 10%–15% smaller in females (Sanchis-Segura et al., 2020). Consequently, studies have proposed methods for careful control of TIV differences between the sexes. One such method is TIV-matching, where males and females of equivalent TIV are matched for the development set (Ebel et al., 2023; Wiersch et al., 2023). With a large sample size, these studies have managed to maintain a high accuracy using traditional ML techniques (Support Vector Machine and Logistic Regression). Another approach involves preprocessing steps, performing spatial non-linear and *z*-score normalization, which reduces TIV bias to some extent, though not entirely (Ebel et al., 2023).

**Figure 1:**
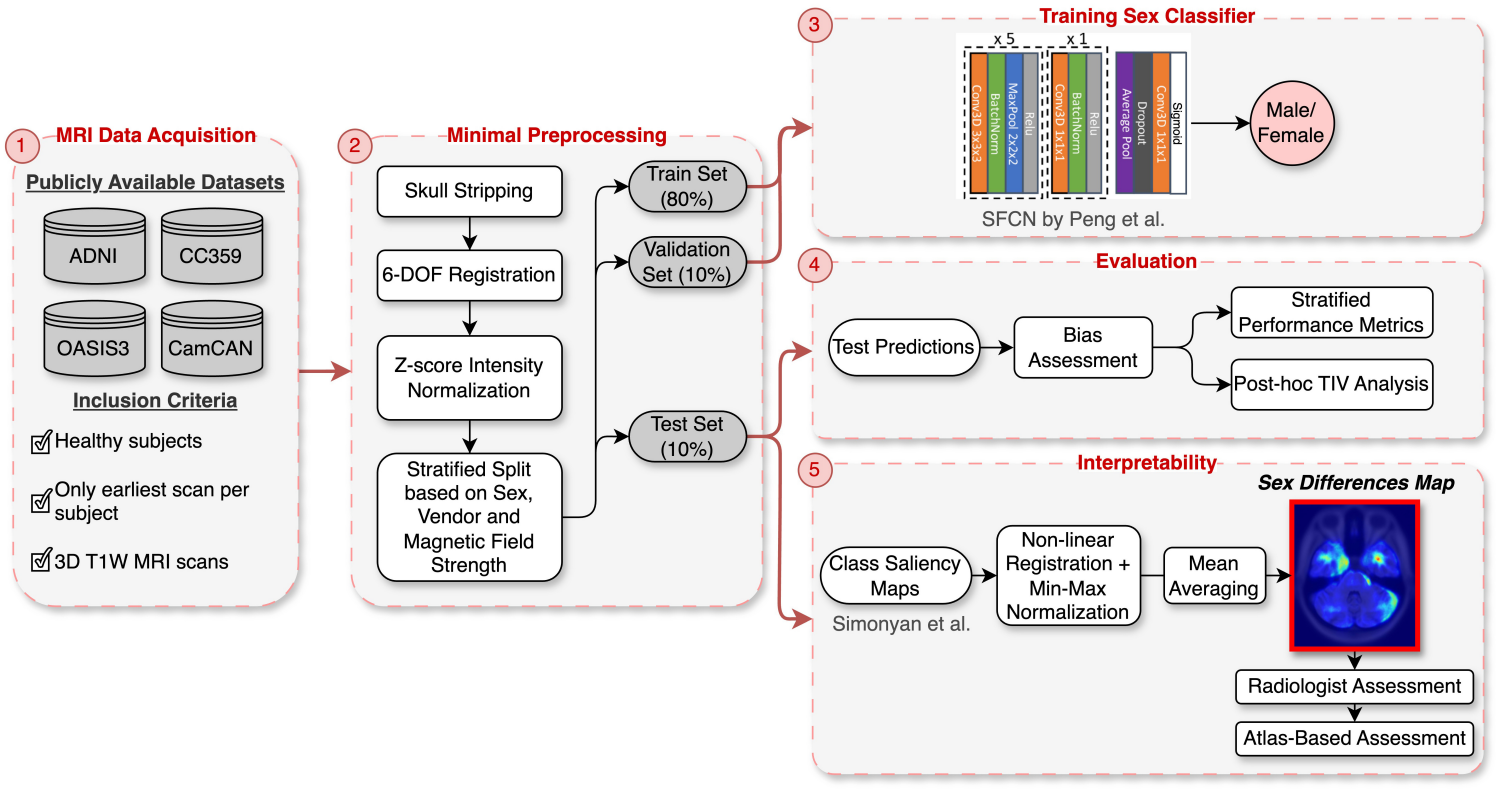
Overview of the methodology: (1) Data acquisition from four publicly available datasets (ADNI, OASIS3, CC359, CamCAN). (2) Minimal preprocessing, including skull stripping, rigid registration, and intensity normalization, followed by stratified splitting based on sex, vendor, and magnetic field strength. (3) Training of the SFCN architecture on the stratified data split. (4) Evaluation with stratified performance metrics and *post-hoc* TIV analysis to assess bias. (5) Interpretability using class saliency maps processed into a “Sex Differences Map” and validated through radiologist and CerebrA atlas assessments.

A key focus of this study is the development of “sex differences map” through saliency analysis, which reveals the critical brain regions involved in distinguishing between male and female brains. These maps provide valuable insights into neuroanatomical distinctions between the sexes and hold potential for guiding the development of more fair and unbiased AI models for healthcare. To create these maps, we developed a DL model for sex classification using four public brain MRI datasets, applying minimal preprocessing, including skull stripping and rigid registration, to preserve brain structure integrity. We utilized a 3D Convolutional Neural Network (CNN) architecture for this task. Instead of controlling for TIV during training, we opted to minimally preprocess the data and later conducted a *post-hoc* analysis to determine whether the model primarily utilized TIV or successfully identified other critical neuroanatomical features for sex classification. A key component of our methodology was the integration of explainability techniques to identify the brain regions that the DL model deemed important for distinguishing between male and female brains. We assessed these saliency maps both quantitatively, using a region-labeled atlas, and qualitatively, through expert evaluation by a radiologist, ensuring that the model's focus aligned with relevant neuroanatomical structures. Figure 1 provides an overview of the methodology steps.

Different from related works in the literature, we opted not to apply TIV adjustment techniques, such as TIV-matching, during the training phase of this study. While TIV adjustment can help control for volumetric differences between male and female brains, these techniques often require discarding valuable data or performing extensive preprocessing. Such preprocessing can introduce artifacts or lead to the loss of critical neuroanatomical information, particularly in studies focusing on small or intricate brain structures. This loss of detail can potentially obscure relevant features and limit the overall performance of deep learning models. Given these concerns, we decided to retain the natural variation in our datasets and perform minimal preprocessing, which allowed the model to learn directly from a broader range of brain data. *Post-hoc* analysis was conducted to assess the model's reliance on TIV, ensuring that it captured relevant neuroanatomical features beyond simple volumetric differences.

The contributions of this study are as follows: (1) Creation of a “sex differences map” through class saliency analysis, identifying brain regions crucial for distinguishing male and female brains, validated through both quantitative analysis and radiologist evaluation; (2) Adaptation of a 3D CNN architecture for sex classification using publicly available brain MRI datasets with minimal preprocessing to preserve brain structure integrity; (3) Detailed evaluation of model performance, including *post-hoc* analysis of its reliance on TIV, and detailed performance metrics stratified by datasets, vendors, magnetic field strengths, and age range to assess robustness and identify potential biases.

## 2 Methodology

### 2.1 Datasets

For the experiments, 3D T1-weighted MR images from four publicly accessible datasets were employed: the Calgary-Campinas-359 (CC-359) dataset (Souza et al., 2018), the Open Access Series of Imaging Studies-3 (OASIS-3) (LaMontagne et al., 2019), the Alzheimer's Disease Neuroimaging Initiative (ADNI) (Jack Jr et al., 2008), and the Cambridge Center for Aging and Neuroscience dataset (Cam-CAN) (Shafto et al., 2014; Taylor et al., 2017). The inclusion of these datasets was driven by the need for healthy control groups, leading us to aggregate data from multiple sources. Additionally, the availability of sex and age information was a critical criterion for dataset selection. Participant demographics in these datasets were determined through self-reporting. In instances where multiple scans were available for a subject, only their earliest scan was used to avoid data leakage. Table 1 summarizes the information available for each dataset, including sex, age, vendor, and magnetic field strength. Notably, these datasets represent females and males almost equally.

**Table 1:** Dataset description.

**Dataset**	**#subjects**	**Sex (F:M)**	**Age**	**Vendor**	**Field (T)**
CC-359	359	183:176	29–80	GE, Philips, Siemens	1.5, 3
ADNI	367	186:181	56–90	GE, Philips, Siemens	1.5, 3
OASIS-3	731	416:315	42–95	Siemens	1.5, 3
CamCAN	653	330:323	18–88	Siemens	3

Columns from left to right: dataset name, number of subjects (all healthy), sex distribution, age range, MRI vendor, and magnetic field strength.

### 2.2 Preprocessing

The pre-processing approach was deliberately kept minimal to standardize the heterogeneous multi-center datasets without significantly altering their original state. Minimal preprocessing preserves the natural neuroanatomical features crucial for tasks like sex classification while ensuring the data is adequately standardized for DL training (Grødem et al., 2024). The specific steps taken, including brain extraction, rigid registration, and intensity normalization, are standard practices in brain MRI preprocessing (Glasser et al., 2013).

To implement this minimal approach, the initial step in this process involved brain extraction of three datasets using the SynthStrip tool from Freesurfer (skull stripping masks are available for CC359) (Hoopes et al., 2022; Kelley et al., 2024). Subsequently, the brain-extracted images were registered to the MNI152 standard atlas using FSL's FLIRT tool (Smith et al., 2004). Six-degree-of-freedom rigid registration was chosen to rotate and translate images to a common space without altering individual brain volumes. The processed scans were of size (193, 229, 193), and *z*-score normalization was applied to image intensities by subtracting the mean and dividing by the standard deviation of the voxel intensities. The processed images have a mean intensity of 0, standard deviation of 1, and a voxel size of 1 mm^3^. TIV statistics for the raw images in the test set were estimated using the Freesurfer “recon-all” command tool. The estimated TIVs were in *mm*^3^ and were divided by 1,000 to obtain measurements in ml (Sanchis-Segura et al., 2022).

### 2.3 Sex classifier

To perform the sex classification task, we utilized a CNN architecture, the Simple Fully Convolutional Network (SFCN) (Peng et al., 2021). Although this model was proposed for brain age prediction as a classification task, it also showed promising performance in sex classification. This architecture comprises seven blocks in total: the first five blocks each consist of a 3 × 3 × 3 convolutional layer, batch normalization layer, a max pooling layer, and ReLU activation to generate the feature map and reduce the spatial dimension. The sixth block has a 1 × 1 × 1 convolutional layer, a batch normalization layer, and ReLu activation. The last block contains an average pooling layer, a dropout layer (50% during training), and a convolutional layer. The model's architecture was largely retained as the original model with minor changes: the kernel dimensions of the average pooling layer were set to 6 × 7 × 6 to align with the input size, the number of classes was set to 1 for binary classification, and the softmax output activation was replaced with the sigmoid function to better suit the binary nature of the classification task with one output neuron. Unlike softmax, which is designed for multi-class classification, sigmoid outputs the probability of the instance belonging to the positive class (in this case arbitrarily chosen to be the Male class).

### 2.4 Interpretability method

To better comprehend the decision-making process of the DL model, we utilized class saliency maps as proposed by Simonyan et al. (2013). This method involves the computation of the gradient of the class score with respect to the input image layer, using a single back-propagation pass through the image classification network. The gradient indicates the contribution of each voxel to the class score, providing a spatial visualization of the regions within an image that are most important for the model's decision. We chose this method after testing several alternatives, such as GradCAM and SmoothGrad, because it consistently produced the most detailed and informative saliency maps, crucial for analyzing subtle neuroanatomical differences relevant to sex classification. Additionally, the method is computationally efficient, requiring only a single back-propagation pass through the network. The intensity values in the saliency maps were min-max normalized to range from 0 (least important) to 1 (most important) (Saporta et al., 2022).

### 2.5 Experimental design

This study was designed to evaluate the performance of DL, specifically CNNs, for sex classification. We aggregated brain MRI data from four public datasets to explore the sex differences in the brain. The experimental design was carried out in several stages: preprocessing, dataset splitting, training, evaluation, and interpretability assessment (Figure 1).

Preprocessing steps, including skull stripping, rigid registration, and intensity normalization, were applied to all data prior to the dataset split. These minimal steps were chosen to preserve as much information from the raw data as possible while standardizing the data for model training. The aggregated dataset was divided into three distinct subsets: 80% was allocated for training (1,688 samples), 10% for validation (211 samples), and 10% for testing (211 samples). This division was executed in a stratified manner, ensuring consistent representation of key features—sex, vendor, and magnetic field strength—across subsets. These characteristics were chosen for stratification because they are known to influence MRI data characteristics. Sex differences in brain structure are central to our study, while variations in vendor and magnetic field strength can introduce systematic differences in image quality and signal, which could impact the model's generalizability and accuracy (Souza et al., 2018). Within each subset, the sex distribution was 53% female and 47% male, with vendor distribution of 11% GE, 8% Philips, and 81% Siemens. Magnetic field strengths were represented by 79% at 3 T and 21% at 1.5 T.

To determine the optimal hyperparameters for training the CNN, we conducted preliminary experiments in which we systematically varied key parameters, such as batch size, initial learning rate, and learning rate scheduler. These experiments involved testing a range of commonly used values and assessing their impact on convergence speed, training and validation loss, and overall accuracy. Based on these preliminary results, we selected the following hyperparameters for the final model training: a batch size of 16, an initial learning rate of 0.01, and the Adam optimizer with Binary Cross Entropy as the loss function (Semenov et al., 2019). To further optimize the learning rate during training, we implemented the “ReduceLROnPlateau” strategy, which decreased the learning rate by a factor of 0.1 after five epochs without improvement in validation loss. Training was conducted over 50 epochs.

To mitigate overfitting and improve model generalization, data augmentation was applied during training. Specifically, 50% of the scans were randomly rotated by 15° on-the-fly. This rotation was chosen to introduce variability in scan orientation without distorting key neuroanatomical structures that are crucial for sex classification. Other augmentation techniques, such as random flipping and contrast adjustments, were considered but ultimately not implemented due to their limited impact during preliminary experiments.

The optimized model with the lowest validation loss during the epochs was selected for evaluation on the test set. Performance was assessed using accuracy, balanced accuracy, recall, precision, and F1-score. To further investigate the model's decision-making process, saliency maps for each correctly classified scan in the test set were generated using the Captum library (Kokhlikyan et al., 2020). Non-linear registration was applied to these maps to align them with the MNI152 standard brain atlas using FSL FNIRT, along with min-max normalization. The processed maps were averaged to identify regions critical for distinguishing between male and female brains. Non linear registration was crucial because the saliency maps were in the native image space of each scan, and variations in brain size and shape across subjects could lead to misalignment during averaging. Therefore, we ensured that corresponding brain regions were aligned across all subjects, allowing for the creation of a single composite map that accurately reflects sex differences in brain structure from the DL model's perspective.

The “sex differences map” was first analyzed by a radiologist to ensure that the model's focus aligned with clinically relevant neuroanatomical structures. After the radiologist's review, we used the CerebrA atlas to quantify the importance of the specific brain regions identified by the model. The CerebrA atlas provides detailed cortical and subcortical labels for the brain (Manera et al., 2020). For each labeled region, a saliency score was calculated, defined as the proportion of salient voxels within that region (voxels with saliency values >0.1). Additionally, the mean saliency value for each region was computed and then min-max normalized to the range [0, 1]. To derive a more representative measure of importance, we calculated a weighted saliency score by multiplying the saliency score by the normalized mean saliency value (Stanley et al., 2022). This approach allowed us to quantify the significance of each brain region in the model's predictions.

### 2.6 TIV considerations

TIV is known to be a potential confounding factor in sex classification models. However, in this study, we deliberately chose not to correct for TIV during the training phase. One of the core objectives of our study is to minimize preprocessing steps in order to preserve the integrity of the brain images and enhance the applicability of DL models in clinical settings. TIV correction represents an extensive preprocessing step that might introduce biases or obscure relevant biological information, particularly in small and subtle brain structures. By avoiding TIV correction, we aim to enable the model to learn directly from minimally processed brain images, thereby preserving the natural variations in brain structures that are potentially crucial for accurate sex classification.

Instead of adjusting for TIV during training, we chose to assess its impact on the model's performance in a *post-hoc* analysis. This approach allows us to determine the extent to which the model depends on TIV versus other neuroanatomical features, offering a clearer understanding of the model's reliance on different aspects of the brain data.

**Figure 2:**
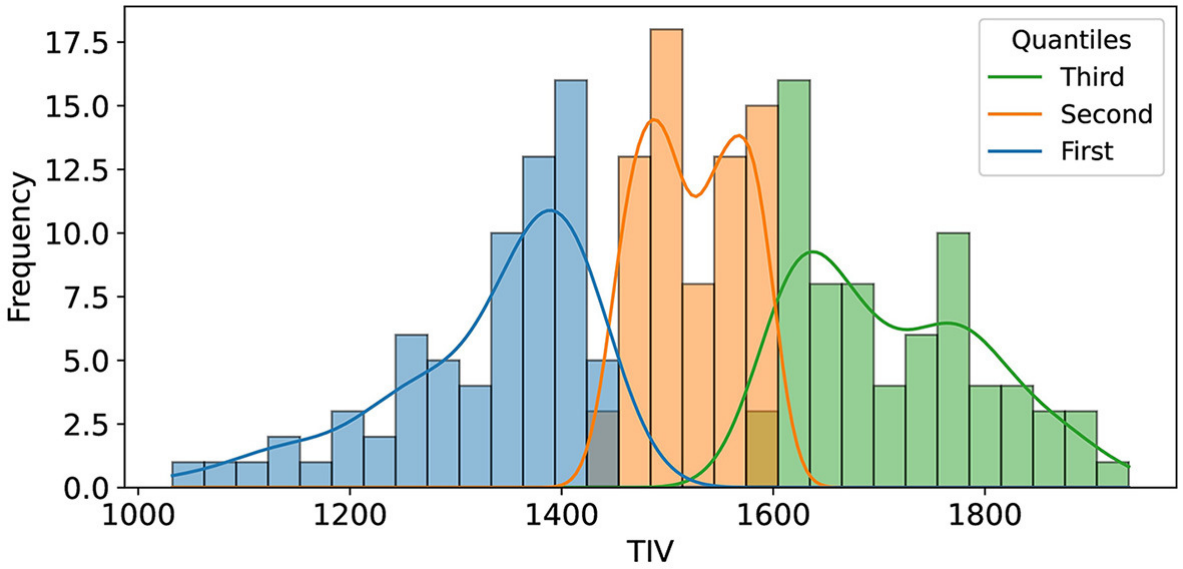
The illustration shows the distribution of Total Intracranial Volume (TIV) across three distinct quantiles, each comprising 70 samples. These quantiles were determined to facilitate an in-depth analysis of model performance variations across varying ranges of TIV.

For TIV analysis, Freesurfer measurements were used, excluding one unprocessable test sample, resulting in 210 samples. To assess the impact of TIV on model performance, we performed a *post-hoc* analysis using TIV quantiles and Kernel Density Estimation (KDE). The test set was divided into three TIV quantiles (each including 70 samples), representing low, medium, and high TIV ranges, to systematically evaluate model performance across different brain sizes (Figure 2). This quantile-based approach ensured that each TIV range was equally represented, allowing us to identify any biases the model might have toward extreme TIV values.

We also employed KDE to focus the analysis on the TIV range where male and female distributions overlapped the most, i.e., where TIV differences were minimized. A 20% density threshold was applied to exclude the lower-density tails of the distribution, thus emphasizing the regions where the TIV values of both sexes were most similar and TIV was not an overt differentiating factor between sexes (Figure 5). This approach, especially effective in handling non-normally distributed data, focuses on the densest regions, avoiding outliers or less representative data. The threshold was selected after testing several alternatives, finding that 20% provided the most informative results without excluding too much data or including too many extreme cases. This approach helped us isolate the TIV ranges that contributed most to balanced classification performance, minimizing potential sex-based bias.

## 3 Results

The model with the lowest validation loss was evaluated using binary classification metrics. This model achieved an accuracy of 89.5% on the validation set. The overall accuracy on the test set was 87.20%, with subgroup accuracies of 88% for females and 86% for males. Overall AUC-ROC, F1-score, precision, and recall were 93.36%, 86.67%, 86.14%, and 87%, respectively.. We conducted a detailed evaluation of model performance across various subgroups based on different datasets, scanner vendors, magnetic field strengths, and age ranges. The performance metrics for each subgroup within these variables are presented in Table 2.

**Table 2:** Comparison of performance metrics across different dataset sources, MRI scanner vendors, and magnetic field strengths in the test set.

**Variable**	**Subgroup**	**Balanced accuracy**	**Precision**	**Recall**	**F1 score**
Dataset	ADNI	0.863	0.846	0.917	0.880
	CC359	0.851	0.929	0.765	0.839
	OASIS3	0.858	0.731	0.905	0.809
	CamCAN	0.920	0.971	0.868	0.917
Vendor	GE	0.917	1.000	0.833	0.909
	Philips	0.819	0.857	0.750	0.800
	Siemens	0.877	0.855	0.888	0.871
Field	1.5T	0.837	0.818	0.857	0.837
	3T	0.885	0.884	0.873	0.878
Age	< 55	0.889	1.000	0.778	0.875
	55–70	0.844	0.788	0.867	0.825
	≥70	0.886	0.870	0.930	0.899

**Figure 3:**
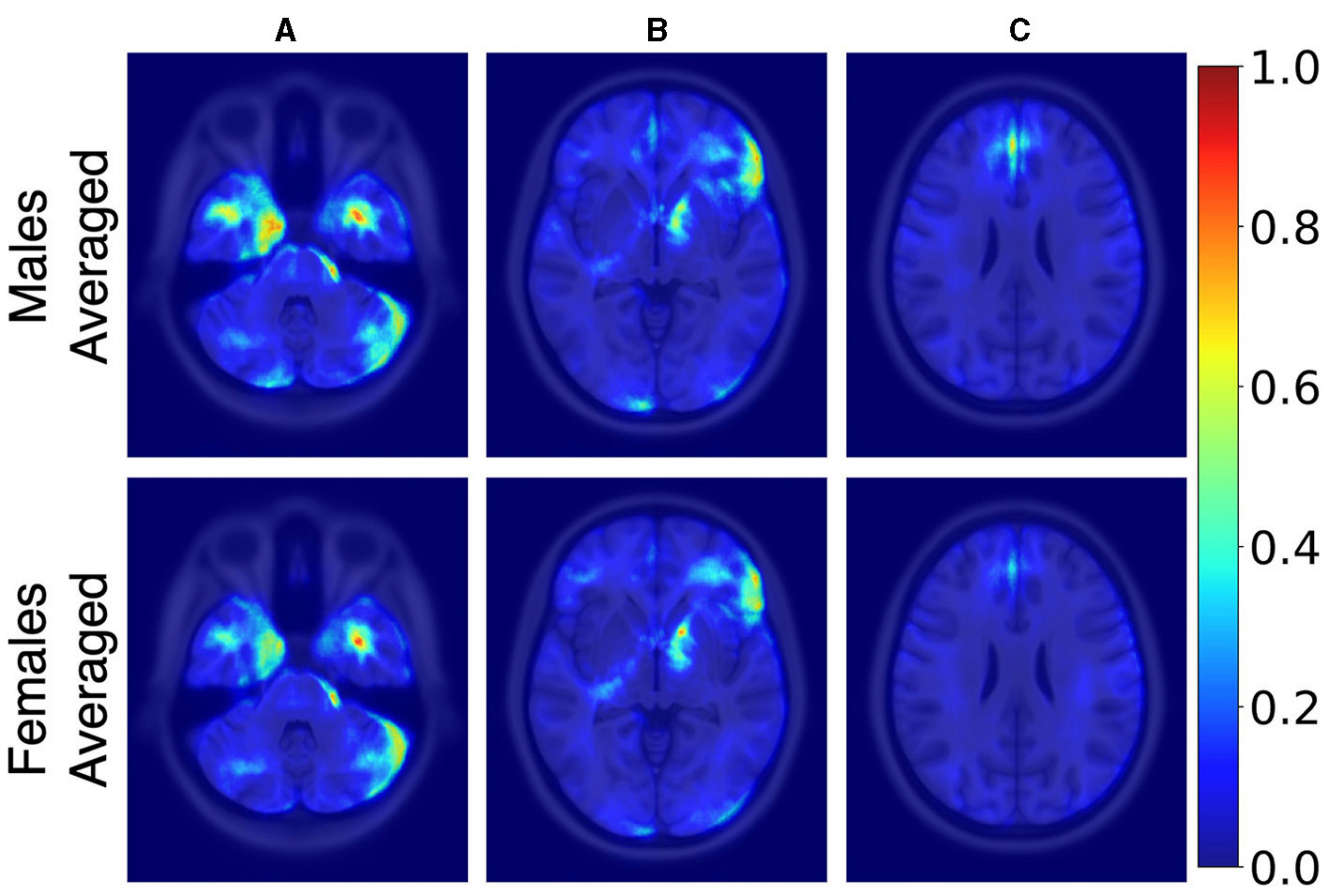
Axial MRI slices comparing average saliency maps for correctly classified male (top row) and female (bottom row) subjects. From left to right, Panels **(A)**, **(B)**, and **(C)** correspond to different axial levels for both male and female subjects. Normalized saliency scores are color-coded from blue (least important, score 0) to red (most important, score 1).

Figure 3 illustrates the averaged saliency maps for males and females, respectively. This map highlights the brain regions deemed significant by the model for making predictions. It was generated by averaging the saliency maps of accurately classified test samples. Correctly classified samples were utilized in order to identify the most consistent and reliable regions identified by the model. The Spearman correlation between these two maps (females vs. males) was 0.99 (*p* < 0.05), suggesting a very strong positive relationship, indicating that the maps consistently rank the importance of regions in a similar manner.

### 3.1 *Post-hoc* TIV analysis

In our *post-hoc* analysis, we observed that the accuracy values across TIV-based quantiles were relatively consistent, ranging from 85 to 89%. However, classification performance varied with TIV; females were classified more accurately at lower TIVs (60 true positives, one false positive), while males were more accurately classified at higher TIVs (59 true positives, one false positive). Interestingly, the middle TIV quantile showed a similar error rate for both sexes. Table 3 summarizes the performance across three TIV quantiles separately for males and females. Balanced accuracy on each TIV quantile highlights the true performance by accounting for the imbalance within the quantiles.

**Table 3:** Model accuracy for sex classification across different Total Intracranial Volume (TIV) quantiles.

**Quantile**	**TIV range**	**Female accuracy**	**Male accuracy**	**Balanced accuracy**
First	1,032–1,443	98.4%	33.3%	65.8%
Second	1,447–1,597	87.2%	80.6%	83.9%
Third	1,599–1,935	30.0%	98.3%	64.2%

Each quantile represents a range of TIV values in milliliters, with separate accuracy percentages reported for female and male subjects, as well as the overall balanced accuracy for each quantile.

**Figure 4:**
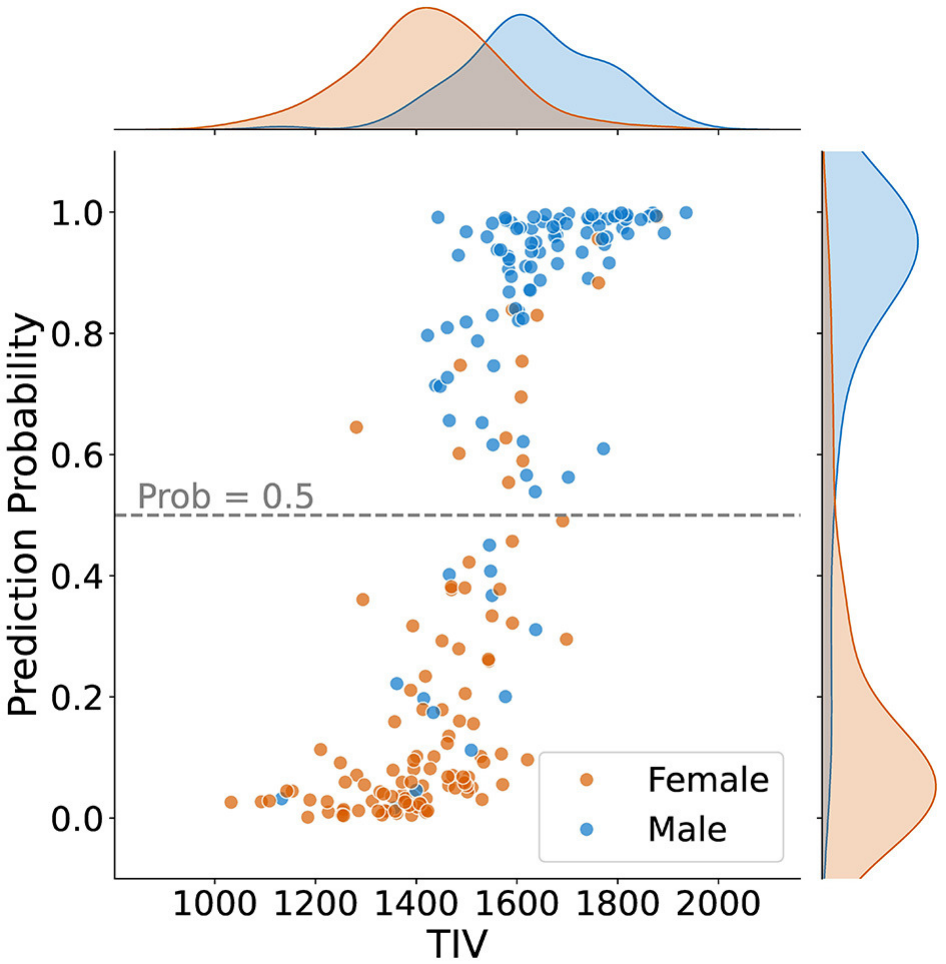
Sex classification prediction according to total intracranial volume (TIV) for 211 samples, including density plots for male and female distributions. The dashed line represents the classification threshold at a probability of 0.5. While the extremes of the TIV scale are strongly correlated with sex prediction–higher TIV for males and lower TIV for females–there is less bias in the middle TIV range.

Figure 4 shows how predictions varied based on TIV values. It can be observed that most samples in the lower TIV range are classified as female, and most scans in the higher TIV range are classified as male. In other words, as TIV increases, the number of scans classified as male increases. In the middle range of TIV, classification as male versus female is balanced, indicating reduced model bias compared to the higher and lower TIV extremes.

**Figure 5:**
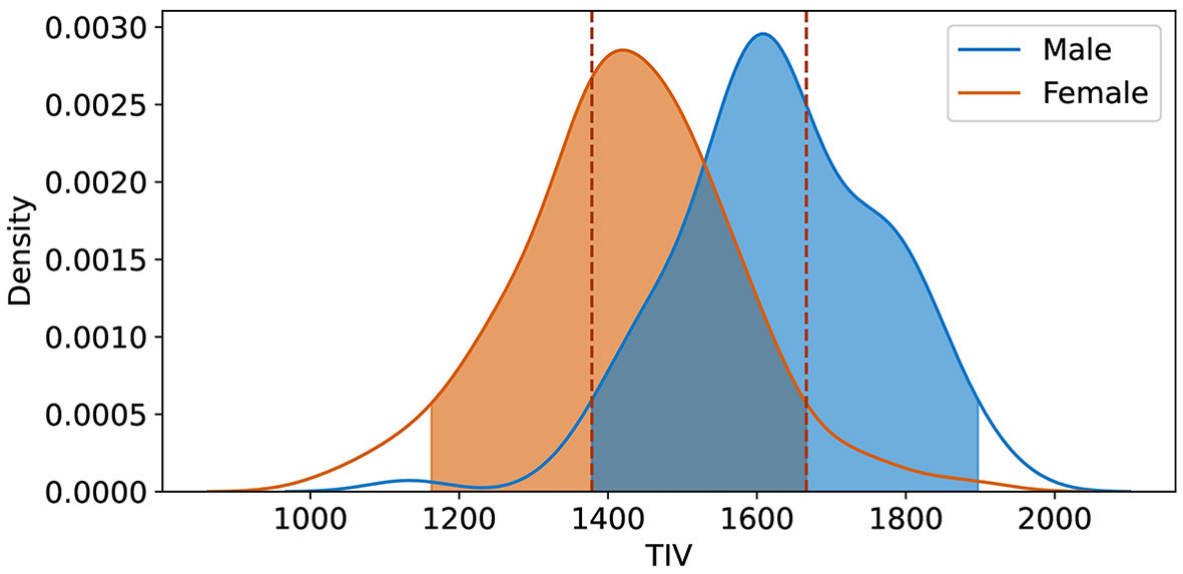
Kernel Density Estimation (KDE) of TIV for female and male groups, with a 20% threshold applied to exclude lower density values. The overlapping region *R*_*tiv*_ = [1, 378ml, 1, 666ml] indicates the range of TIV values most common to both sexes, signifying the highest density overlap between the distributions.

Using KDE with a 20% threshold, we identified the overlapping TIV range of 1,378–1,666 (*R*_*tiv*_) (Figure 5). The model's classification performance was most balanced between sexes in this range. The model's accuracy within this range suggests that the reliance on TIV as a discriminative feature was minimized, resulting in more equitable performance across sexes.

**Figure 6:**
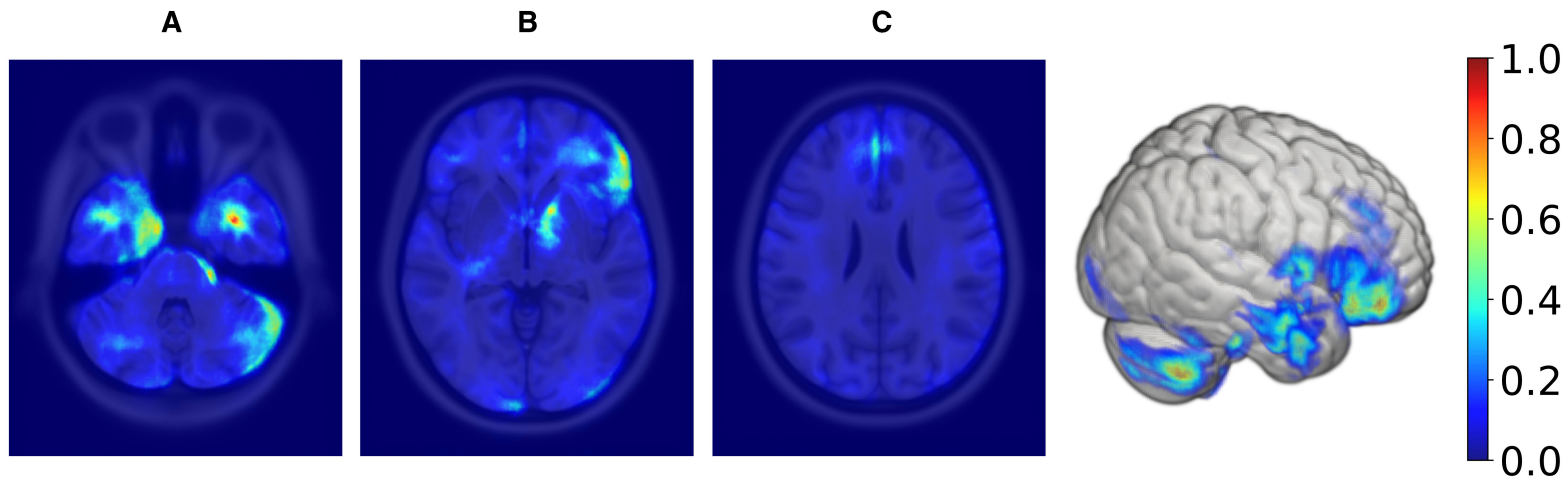
Axial MRI slices and a 3D rendering displaying average saliency map for 98 correctly classified samples (50% females, 50% males) within the TIV range of 1,378–1,666 ml, with blue to red indicating low to high importance. The range was determined using KDE, filtering out values below 20% of maximum density for both sexes. From left to right, Panels **(A)**, **(B)**, and **(C)** highlight different axial levels with key brain structures for sex classification.

In order to create the final averaged saliency map, a balanced subset was extracted from *R*_*tiv*_, comprising an equal number of male and female samples (98 in total). This subset was specifically chosen to include only correctly classified samples. This approach ensures the final map is equally influenced by female and male samples and represents reliable and consistent regions considered important. We computed a final averaged saliency map from this subset, depicted in Figure 6.

Supratentorial regions that were most important for prediction included the inferior frontal lobe and frontal operculum (seen in panel Figure 6B), the parasagittal frontal lobe, more specifically the medial aspect of the superior frontal gyrus (seen in panel Figure 6C), the mesial temporal lobe cortex and subcortical white matter (seen in panel Figure 6A), the anterior limb and genu of the internal capsule (seen in panel Figure 6A) and the optic chiasm and adjacent posterior aspect of the gyrus rectus (not shown). Infratentorial regions that were most important for prediction included the anterior and lateral surface of the pons and mesencephalon (seen in panel Figure 6A) and the cerebellar hemispheric surfaces, particularly the lateral surface (seen in panel Figure 6A).

**Figure 7:**
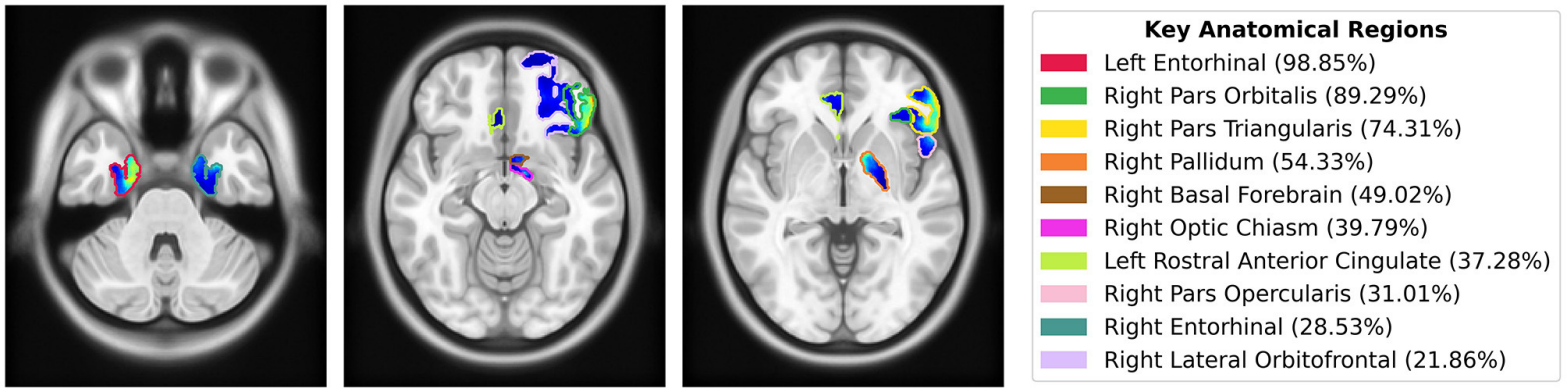
Saliency map overlaid on MNI152 template, highlighting the top 10 anatomical regions from the CerebrA atlas identified by the model using weighted saliency scores. Weighted saliency scores represent each region's importance in the model's predictions, considering both the proportion of salient voxels and their saliency values. The regions are color-coded according to the legend, with percentages indicating the weighted saliency score for each region.

To quantify the importance of brain regions, we computed saliency scores, which represent the percentage of salient voxels within each labeled region from the CerebrA atlas. Additionally, we calculated weighted saliency scores, which incorporate both the proportion of salient voxels and their saliency values, reflecting the significance of each region in the model's predictions. Detailed results for all regions are provided in the Supplementary Table S1. The top 10 regions identified through this analysis, ranked by weighted saliency scores, include the left entorhinal cortex, right pars orbitalis, right pars triangularis, right pallidum, right basal forebrain, right optic chiasm, left rostral anterior cingulate, right pars opercularis, right entorhinal cortex, and right lateral orbitofrontal. Figure 7 visualizes the saliency map overlaid on these top anatomical regions identified by the model along with their corresponding weighted saliency scores.

## 4 Discussion

The proposed sex classification model achieved an accuracy of 87% on the test set. We deliberately chose not to apply TIV adjustment techniques, such as TIV matching, which often results in the exclusion of a significant portion of the available dataset. Additionally, we did not apply preprocessing steps like non-linear spatial and *Z*-Score normalization. By minimizing the use of preprocessing, we aimed to keep the brain structures and features as close to their natural form as possible, avoiding the introduction of artifacts or noise into the images.

**Figure 8:**
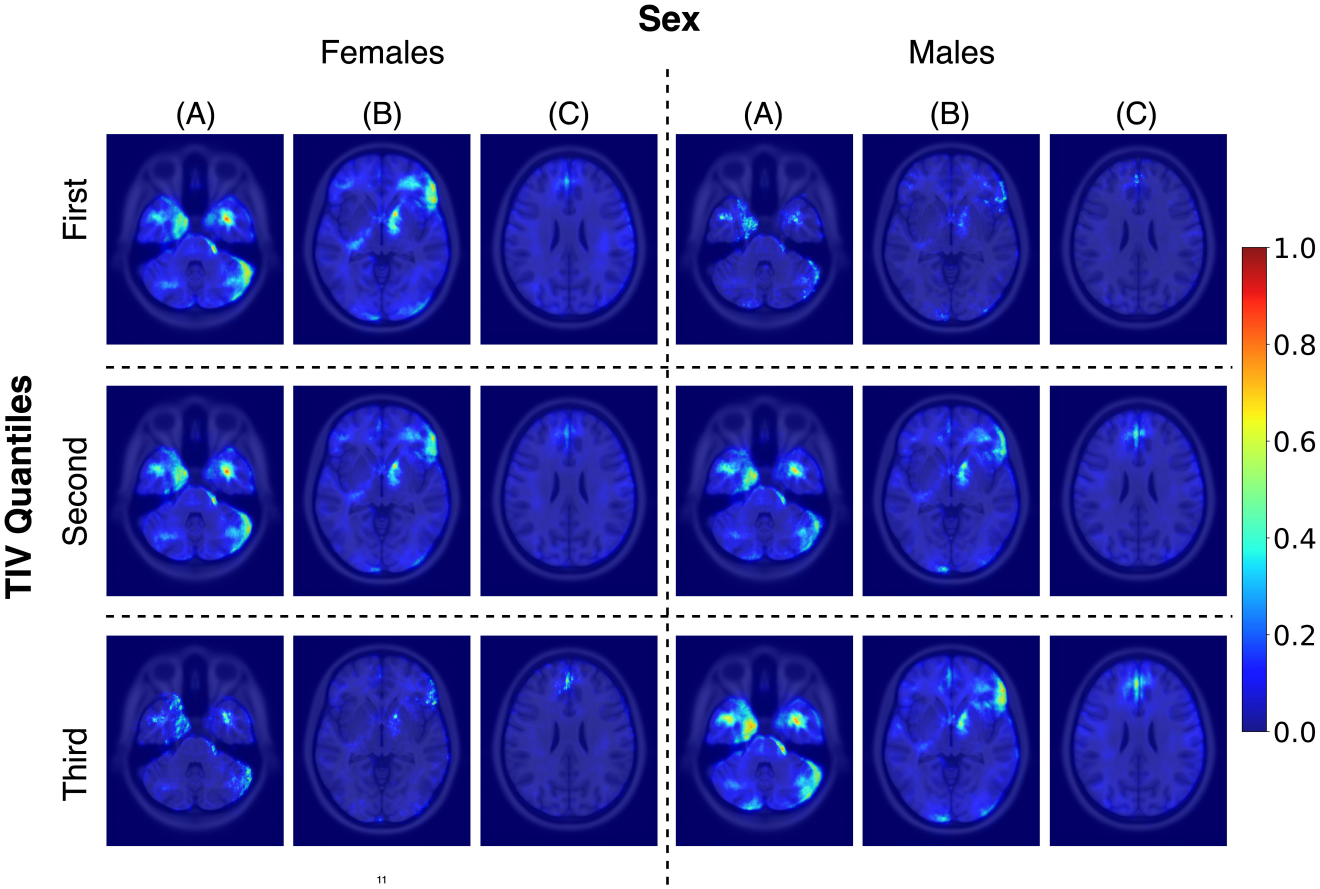
Average saliency maps for correctly classified female and male subjects across three TIV quantiles, each representing a specific range of TIV values. Panels **(A)**, **(B)**, and **(C)** showcase axial levels that highlight key brain structures relevant for sex classification.

While TIV correction is a common practice, it may not always be necessary or desirable depending on the study's objectives. Given our goal of developing models that are more generalizable and less dependent on specific preprocessing steps, avoiding TIV correction was a considered and valid choice. In the *post-hoc* analysis of TIV, a comparison of model performance across TIV values indicated that predictions were less biased in *R*_*tiv*_ where the TIV distribution of females and males had the most overlap (Figure 4). The model's accuracy in *R*_*tiv*_ was comparable to its overall accuracy. Moreover, the averaged saliency maps over all test samples (Figure 3) and the balanced subset in *R*_*tiv*_ (Figure 6) showed similar regions with a Spearman correlation of 0.99 (*p* < 0.05). Figure 8 further confirms consistent predictive regions across TIV quantiles and sexes.

This study advances sex classification using brain MRI data by employing a 3D CNN model with minimal preprocessing, intentionally avoiding TIV adjustments to maintain natural brain structure variations. Unlike studies such as Ebel et al. (2023) and Wiersch et al. (2023), which control for brain size to reduce bias, our approach embraces these variations to explore their relevance in clinical settings. Ebel et al. (2023) used logistic regression on volume-matched gray matter data, achieving 92% accuracy, and a 3D CNN (BraiNN) on the same data, achieving 88% accuracy. Wiersch et al. (2023) utilized voxel-wise gray matter volume with an SVM, reaching 92.55% accuracy after TIV control. In contrast, Hu et al. (2019) focused on optimizing their multi-layer 3D convolutional extreme learning machine (MCN-ELM), achieving 98.06% accuracy without TIV adjustment, but they did not emphasize interpretability or the identification of brain regions contributing to sex differences.

Our minimal preprocessing allows the identification of nuanced sex differences, validated through interdisciplinary radiological assessment. While Bozek et al. (2021) also utilized interpretability using SHAP analysis on volumetric features, our approach extends this by leveraging 3D MRI images to create a detailed “sex differences map” through saliency analysis. This not only provides a high classification accuracy of 87% but also offers a robust, interpretable map of sex differences, validated by experts. To our knowledge, no other study has focused as extensively on visualizing important brain regions for sex classification.

Upon reviewing the saliency maps, Radiologist J. Ospel (co-author) observed that key regions for sex prediction in our model, such as the mesial temporal lobe cortex and superior frontal gyrus cortex, are situated in the gray matter. This observation aligns with previous studies showing notable differences in gray matter volumes between males and females, even when total brain volume is accounted for Liu et al. (2020). Gray matter predominantly contains the somatodendritic tissue of neurons (i.e., the actual cell bodies), whereas white matter is primarily comprised of myelinated connecting axons (the “connecting fibers”). Some studies have shown a relatively higher percentage of gray matter in women, and it has been hypothesized that this results in prioritization of computational power over transfer across distant regions and could compensate for the overall lower brain parenchymal volume in women. Interestingly, not only total gray matter volumes, but also the distribution of gray matter between the cerebral hemispheres, have been shown to differ between women and men, with women showing a more symmetric distribution between both hemispheres (Gur et al., 1999).

Of note, regions like the cerebellum and temporal lobe cortex, known for their sex differences in gray matter volume (Lotze et al., 2019), were also significant in our model. Moreover, our model identified areas like the internal capsule and optic chiasm/posterior gyrus rectus, which, to our knowledge, have not been previously highlighted as being different among sexes in the literature. These regions have distinct functions: the internal capsule is crucial for connecting the cerebral hemispheres with various subcortical structures, the optic chiasm is part of the optic pathway and contains the crossing fibers of both optic nerves—lesions that compress the optic chiasm such as pituitary adenomas, often lead to homonymous hemianopsia, i.e., visual field loss in the same halves of the visual field of each eye (Wolberg et al., 2024). Other regions, such as the inferior frontal gyrus, have more complex roles in inhibitory and attention control, and language production, depending on hemisphere dominance (Hampshire et al., 2010; Ishkhanyan et al., 2020).

Quantitative analysis using the CerebrA atlas further supported our findings. For each labeled region, we calculated a saliency score, representing the proportion of salient voxels, and a weighted saliency score that accounted for voxel intensity. These scores quantified the importance of each brain region in the model's predictions. The top regions identified through this analysis included the left and right entorhinal cortex (part of the medial temporal lobe), right pars orbitalis, right pars triangularis, right pars opercularis, and right lateral orbitofrontal cortex (regions in the inferior frontal lobe), as well as the right optic chiasm. These findings align with the radiologist's observations and provide a more detailed understanding of the neuroanatomical areas contributing to our model's decision-making. Additionally, the quantitative analysis highlighted other regions, such as the right pallidum, right basal forebrain, and left rostral anterior cingulate (Figure 7).

Given the association of structural brain anatomy with functionality and behavioral patterns, one might hypothesize that sex differences in brain structure also translate into differences in functionality and behavior. However, this is not necessarily true. For example, while females have brain volumes that are on average 10%–15% smaller compared to males, there is no difference in average intelligence levels between the two sexes (Burgaleta et al., 2012). This highlights the fact that brain size or structure alone does not directly correlate with cognitive performance or behavior.

There is also growing recognition of the numerous compensatory mechanisms and redundancies in the brain that mitigate the impact of anatomical differences on function. Evolutionary processes, hormonal influences, and developmental factors all contribute to shaping brain structure, but these factors do not always lead to functional disparities (DeCasien et al., 2022). Therefore, caution should be taken when inferring sex differences in functionality and behavior based solely on structural differences, as observed anatomical variations may not necessarily translate into functional or behavioral differences between men and women.

We assessed the model's generalization by aggregating multiple public datasets from various institutions. This strategy increased data volume and introduced a range of patient demographics and clinical practices, enriching our dataset's diversity. Including different vendors and magnetic field strengths further diversified our dataset, which is crucial for developing robust DL models adaptable to various clinical settings. Table 2 confirms this, showing consistent performance across subgroups for each variable and reinforcing the model's reliability across diverse clinical scenarios. These measures collectively boost accuracy and reliability, improving real-world healthcare applicability (Rouzrokh et al., 2022).

Sex differences in brain structure are known to interact with aging, which can influence the performance of classification models across different age groups. Research has shown that while sex-related structural differences are present throughout adulthood, they may be more prominent in early to mid-adulthood (Lotze et al., 2019). We analyzed model performance across different age ranges using thresholds based on key periods of brain aging identified in the literature. The first threshold was set at 55 years, as studies indicate that this is the period when more pronounced age-related changes in brain structure, particularly in gray and white matter, begin to emerge, marking the transition from midlife to older adulthood (Raz et al., 2005). The second threshold was set at 70 years, reflecting the transition into advanced aging, where the rate of brain atrophy accelerates significantly, particularly in regions such as the prefrontal cortex and hippocampus (Zhang et al., 2010). These thresholds allow us to explore how aging might impact sex classification model performance, capturing both midlife transitions and advanced aging effects. As shown in Table 2, the model performed consistently across all age groups, with balanced accuracy values of 0.889 for individuals under 55, 0.844 for those between 55 and 70, and 0.886 for those over 70. While there is some variation in performance, the model's ability to classify sex is generally stable across these age ranges. The slight dip in performance in the 55–70 age group could reflect the onset of age-related changes that modestly affect the structural features the model relies on, but the overall consistency suggests that sex classification remains effective across different age ranges.

Many brain disorders have a clear sex prevalence. As an example, demyelinating diseases such as multiple sclerosis and other immune-mediated inflammatory brain disorders are more prevalent in females (Avila et al., 2018), while certain types of neurodegenerative diseases, such as Parkinson's disease, occur more commonly in males (Young et al., 2023). This implies that the pre-test probability for many neurological conditions [the probability of a screened person having the disease (Attia et al., 2004)] is influenced by patient sex, and accurate classification of patient sex would therefore be expected to improve any algorithm aimed at diagnosing this condition. Furthermore, for some brain conditions, the prognosis differs between males and females. For example, after being diagnosed with glioblastoma, the most aggressive of the primary brain tumors, males have, on average, shorter survival compared to females (Moore et al., 2022).

Furthermore, sex differences in brain atrophy patterns offer another example of how these variations might inform individualized treatment strategies. For instance, for Alzheimer's disease, women often experience a faster atrophy rate in key brain regions compared to men, even with similar biomarker levels. These morphological differences could help tailor treatments more precisely for men and women, moving toward a more personalized medicine approach in AD (Ferretti et al., 2018). Incorporating sex-related information into diagnostic and prognostic models would improve the tool's reliability, supporting fairer and more effective treatment strategies.

To enhance bias mitigation and model optimization using the sex-differences saliency map, we propose two strategies. First, for post-processing bias mitigation, we suggest evaluating model performance through an interpretability map on the test set. If this map shows a strong correlation with the sex-differences saliency map, it could indicate the presence of sex bias within the model. Recognizing such biases is crucial for initiating corrective actions. Second, for in-processing bias mitigation during training, we recommend adapting the reweighing bias mitigation algorithm (Kamiran and Calders, 2012) at a voxel level. This involves adjusting voxel weights in images based on the sex-differences saliency map to direct the model's focus toward more task-relevant brain features and reduce the impact of sex-specific characteristics, thereby promoting a more equitable predictive process.

Our study has limitations, particularly in distinguishing between sex and gender in medical research. Sex, categorized as male or female, refers to biological factors and is considered static. In contrast, gender encompasses social roles, behaviors, expressions, and identity, existing along a continuum and subject to change over time. While gender identity usually aligns with biological sex, it's important to note that transgender individuals, though a small percentage [ < 1% in the U.S. (Chan, 2019)], are part of the population. Therefore, some differences identified by our algorithm might be attributable to gender rather than sex. Additionally, the included datasets presented self-reported sex information, which, for a few individuals, may reflect gender identity rather than sex assigned at birth. This practice is common in biomedical research, where self-report questions are often used to characterize sex or gender identity, and the distinction between these concepts is not always made clear (Stites et al., 2023). This lack of clarity can introduce variability in datasets, as research participants may reference different concepts when answering questions about sex or gender. While we do not expect this issue to significantly impact the development of our algorithm, it is a potential source of variability that should be acknowledged.

Another limitation of our study is related to the class saliency map method. While this method provided the best results in our experiments and the generated maps were validated by our radiologist, it is important to acknowledge that it is sensitive to noise, which can result in less stable visualizations. The effectiveness of this method is closely tied to the CNN architecture, potentially limiting its ability to fully capture non-linearities in deeper models.

Lastly, although we employed stratified splitting in our study to ensure representativeness, we recognize that the use of stratified cross-validation could further improve the assessment of our model's generalizability. Furthermore, we acknowledge that not employing Intensity Standardization techniques (Nyúl et al., 2000) may limit our ability to fully correct for inter-scanner variability in intensity distributions. We applied *z*-score normalization; however, this approach does not account for the non-standardness inherent in MRI intensities across different scanners, which can result in residual discrepancies affecting comparability and generalizability. Incorporating intensity standardization in future work could help address these limitations and improve consistency across datasets.

## 5 Conclusion

A key outcome of our study is the development of sex-differences maps through saliency analysis, which identified critical brain regions involved in sex classification. These maps offer deep learning-based insights into sex differences in the brain and hold the potential to enhance diagnostic and prognostic algorithms by identifying and addressing sex-based biases, ultimately contributing to more equitable healthcare interventions. This interdisciplinary approach, combining machine learning and radiological expertise, emphasizes the importance of fairness in ensuring that the outcomes are not only accurate but also reliable and relevant. Our sex classification model achieved an accuracy of 87%, aggregating four diverse datasets with minimal preprocessing to maintain brain structure integrity. The model showed biases at extreme brain sizes but was less biased in ranges where male and female Total Intracranial Volume distributions overlapped the most.

The insights gained from the sex-differences maps also have broader applicability. For example, these maps could be utilized for the refinement of diagnostic tools for neurological conditions for both males and females by informing critical brain regions. Additionally, they could be adapted to other fields where understanding region-specific influences is important, such as targeted medical interventions based on brain imaging data. While promising, our approach has certain limitations, including the sensitivity of saliency maps to noise, which may affect the stability of the visualizations. Future work could focus on refining these maps to enhance their robustness and exploring their application in different contexts to further validate their utility.

## Data Availability

Publicly available datasets were analyzed in this study. The CC359 dataset is available for download at https://www.ccdataset.com/download. The Cambridge Centre for Ageing and Neuroscience (CamCAN) dataset can be accessed at https://camcan-archive.mrc-cbu.cam.ac.uk/dataaccess/. Data from the Alzheimer's Disease Neuroimaging Initiative (ADNI) is available at https://adni.loni.usc.edu/data-samples/access-data/. The Open Access Series of Imaging Studies (OASIS-3) dataset can be accessed at https://www.oasis-brains.org/#data. The code and reported saliency maps are available at https://github.com/mahsadibaji/sex-differences-brain-dl.

## References

[B1] Attia J. R. Sibbritt D. W. Ewald B. D. Nair B. R. Paget N. S. Wellard R. F.. (2004). Generating pre-test probabilities: a neglected area in clinical decision making. Med. J. Aust. 180, 449–454. 10.5694/j.1326-5377.2004.tb06020.x 15115422

[B2] Avila M. Bansal A. Culberson J. Peiris A. N. (2018). The role of sex hormones in multiple sclerosis. Eur. Neurol. 80, 93–99. 10.1159/000494262 30343306

[B3] Bozek J. Kesedzic I. Novosel L. Bozek T. (2021). Classification and feature analysis of the human connectome project dataset for differentiating between males and females. Automatika 62, 109–117. 10.1080/00051144.2021.188589024123412

[B4] Burgaleta M. Head K. Álvarez-Linera J. Martínez K. Escorial S. Haier R.. (2012). Sex differences in brain volume are related to specific skills, not to general intelligence. Intelligence 40, 60–68. 10.1016/j.intell.2011.10.006

[B5] Chan P. S. (2019). Invisible gender in medical research. Circ. Cardiovasc. Qual. Outcomes. 12:e005694. 10.1161/CIRCOUTCOMES.119.005694 30950649 PMC6457361

[B6] Chouldechova A. Roth A. (2020). A snapshot of the frontiers of fairness in machine learning. Commun. ACM 63, 82–89. 10.1145/3376898

[B7] DeCasien A. R. Guma E. Liu S. Raznahan A. (2022). Sex differences in the human brain: a roadmap for more careful analysis and interpretation of a biological reality. Biol. Sex Differ. 13:43. 10.1186/s13293-022-00448-w 35883159 PMC9327177

[B8] Dibaji M. Gianchandani N. Nair A. Singhal M. Souza R. Bento M.. (2023). “Studying the effects of sex-related differences on brain age prediction using brain MR imaging,” in Workshop on Clinical Image-Based Procedures (Cham: Springer), 205–214. 10.1007/978-3-031-45249-9_20

[B9] Ebel M. Domin M. Neumann N. Schmidt C. O. Lotze M. Stanke M.. (2023). Classifying sex with volume-matched brain MRI. Neuroimage Rep. 3:100181. 10.1016/j.ynirp.2023.100181 PMC12172721 40567385

[B10] Ferretti M. T. Iulita M. F. Cavedo E. Chiesa P. A. Schumacher Dimech A. Santuccione Chadha A.. (2018). Sex differences in Alzheimer disease—the gateway to precision medicine. Nat. Rev. Neurol. 14, 457–469. 10.1038/s41582-018-0032-9 29985474

[B11] Glasser M. F. Sotiropoulos S. N. Wilson J. A. Coalson T. S. Fischl B. Andersson J. L.. (2013). The minimal preprocessing pipelines for the human connectome project. Neuroimage 80, 105–124. 10.1016/j.neuroimage.2013.04.127 23668970 PMC3720813

[B12] Grødem E. O. Leonardsen E. MacIntosh B. J. Bjørnerud A. Schellhorn T. Sørensen Ø.. (2024). A minimalistic approach to classifying Alzheimer's disease using simple and extremely small convolutional neural networks. J. Neurosci. Methods 411:110253. 10.1016/j.jneumeth.2024.110253 39168252

[B13] Gur R. C. Turetsky B. I. Matsui M. Yan M. Bilker W. Hughett P.. (1999). Sex differences in brain gray and white matter in healthy young adults: correlations with cognitive performance. J. Neurosci. 19, 4065–4072. 10.1523/JNEUROSCI.19-10-04065.1999 10234034 PMC6782697

[B14] Hampshire A. Chamberlain S. R. Monti M. M. Duncan J. Owen A. M. (2010). The role of the right inferior frontal gyrus: inhibition and attentional control. Neuroimage 50, 1313–1319. 10.1016/j.neuroimage.2009.12.109 20056157 PMC2845804

[B15] Hoopes A. Mora J. S. Dalca A. V. Fischl B. Hoffmann M. (2022). Synthstrip: skull-stripping for any brain image. Neuroimage 260:119474. 10.1016/j.neuroimage.2022.119474 35842095 PMC9465771

[B16] Hu D. Luo Z. Zhao L. (2019). Gender identification based on human brain structural MRI with a multi-layer 3d convolution extreme learning machine. Cogn. Computa. Syst. 1, 91–96. 10.1049/ccs.2018.0018

[B17] Ishkhanyan B. Michel Lange V. Boye K. Mogensen J. Karabanov A. Hartwigsen G.. (2020). Anterior and posterior left inferior frontal gyrus contribute to the implementation of grammatical determiners during language production. Front. Psychol. 11:685. 10.3389/fpsyg.2020.00685 32395113 PMC7197372

[B18] Jack Jr C. R. Bernstein M. A. Fox N. C. Thompson P. Alexander G. Harvey D. C.. (2008). The Alzheimer's disease neuroimaging initiative (Adni): MRI methods. J. Magn. Reson. Imaging 27, 685–691. 10.1002/jmri.21049 18302232 PMC2544629

[B19] Kamiran F. Calders T. (2012). Data preprocessing techniques for classification without discrimination. Knowl. Inf. Syst. 33, 1–33. 10.1007/s10115-011-0463-8

[B20] Kelley W. Ngo N. Dalca A. V. Fischl B. Zöllei L. Hoffmann M. (2024). Boosting skull-stripping performance for pediatric brain images. arXiv [Preprint]. arXiv:2402.16634. 10.48550/arXiv.2402.16634 39371473 PMC11451993

[B21] Klingenberg M. Stark D. Eitel F. Budding C. Habes M. Ritter K.. (2023). Higher performance for women than men in MRI-based Alzheimer's disease detection. Alzheimers Res. Ther. 15:84. 10.1186/s13195-023-01225-6 37081528 PMC10116672

[B22] Kokhlikyan N. Miglani V. Martin M. Wang E. Alsallakh B. Reynolds J.. (2020). Captum: a unified and generic model interpretability library for pytorch. arXiv [Preprint]. arXiv:2009.07896. 10.48550/arXiv.2009.07896

[B23] LaMontagne P. J. Benzinger T. L. Morris J. C. Keefe S. Hornbeck R. Xiong C.. (2019). Oasis-3: longitudinal neuroimaging, clinical, and cognitive dataset for normal aging and Alzheimer disease. medRxiv. 10.1101/2019.12.13.19014902. [Epub ahead of print].35187166

[B24] Liu S. Seidlitz J. Blumenthal J. D. Clasen L. S. Raznahan A. (2020). Integrative structural, functional, and transcriptomic analyses of sex-biased brain organization in humans. Proc. Nat. Acad. Sci. 117, 18788–18798. 10.1073/pnas.1919091117 32690678 PMC7414084

[B25] Lotze M. Domin M. Gerlach F. H. Gaser C. Lueders E. Schmidt C. O.. (2019). Novel findings from 2,838 adult brains on sex differences in gray matter brain volume. Sci. Rep. 9:1671. 10.1038/s41598-018-38239-2 30737437 PMC6368548

[B26] Manera A. L. Dadar M. Fonov V. Collins D. L. (2020). Cerebra, registration and manual label correction of mindboggle-101 atlas for MNI-ICBM152 template. Sci. Data 7:237. 10.1038/s41597-020-0557-9 32669554 PMC7363886

[B27] Mendrek A. (2015). Is it important to consider sex and gender in neurocognitive studies? Front. Psychiatry 6:83. 10.3389/fpsyt.2015.00083 26082728 PMC4451577

[B28] Moore K. J. Moertel C. L. Williams L. A. (2022). Young adult males have worse survival than females that is largely independent of treatment received for many types of central nervous system tumors: a national cancer database analysis. Cancer 128, 1616–1625. 10.1002/cncr.34120 35132626

[B29] Nyúl L. G. Udupa J. K. Zhang X. (2000). New variants of a method of MRI scale standardization. IEEE Trans. Med. Imaging 19, 143–150. 10.1109/42.836373 10784285

[B30] Peng H. Gong W. Beckmann C. F. Vedaldi A. Smith S. M. (2021). Accurate brain age prediction with lightweight deep neural networks. Med. Image Anal. 68:101871. 10.1016/j.media.2020.101871 33197716 PMC7610710

[B31] Piçarra C. Glocker B. (2023). “Analysing race and sex bias in brain age prediction,” in Workshop on Clinical Image-Based Procedures (Cham: Springer), 194–204. 10.1007/978-3-031-45249-9_19

[B32] Raz N. Lindenberger U. Rodrigue K. M. Kennedy K. M. Head D. Williamson A.. (2005). Regional brain changes in aging healthy adults: general trends, individual differences and modifiers. Cereb. Cortex 15, 1676–1689. 10.1093/cercor/bhi044 15703252

[B33] Rouzrokh P. Khosravi B. Faghani S. Moassefi M. Vera Garcia D. V. Singh Y.. (2022). Mitigating bias in radiology machine learning: 1. data handling. Radiol. Artifi. Intell. 4:e210290. 10.1148/ryai.210290 36204544 PMC9533091

[B34] Sanchis-Segura C. Aguirre N. Cruz-Gómez Á. J. Félix S. Forn C. (2022). Beyond “sex prediction”: estimating and interpreting multivariate sex differences and similarities in the brain. Neuroimage 257:119343. 10.1016/j.neuroimage.2022.119343 35654377

[B35] Sanchis-Segura C. Ibañez-Gual M. V. Aguirre N. Cruz-Gómez Á. J. Forn C. (2020). Effects of different intracranial volume correction methods on univariate sex differences in grey matter volume and multivariate sex prediction. Sci. Rep. 10:12953. 10.1038/s41598-020-69361-9 32737332 PMC7395772

[B36] Saporta A. Gui X. Agrawal A. Pareek A. Truong S. Q. Nguyen C. D.. (2022). Benchmarking saliency methods for chest x-ray interpretation. Nat. Mach. Intell. 4, 867–878. 10.1038/s42256-022-00536-x

[B37] Semenov A. Boginski V. Pasiliao E. L. (2019). “Neural networks with multidimensional cross-entropy loss functions,” in Computational Data and Social Networks: 8th International Conference, CSoNet 2019, Ho Chi Minh City, Vietnam, November 18-20, 2019, Proceedings 8 (Cham: Springer), 57–62. 10.1007/978-3-030-34980-6_5

[B38] Shafto M. A. Tyler L. K. Dixon M. Taylor J. R. Rowe J. B. Cusack R.. (2014). The cambridge centre for ageing and neuroscience (Cam-CAN) study protocol: a cross-sectional, lifespan, multidisciplinary examination of healthy cognitive ageing. BMC Neurol. 14, 1–25. 10.1186/s12883-014-0204-1 25412575 PMC4219118

[B39] Simonyan K. Vedaldi A. Zisserman A. (2013). Deep inside convolutional networks: visualising image classification models and saliency maps. arXiv [Preprint]. arXiv:1312.6034. 10.48550/arXiv.1312.6034

[B40] Singh A. Sengupta S. Lakshminarayanan V. (2020). Explainable deep learning models in medical image analysis. J. Imaging 6:52. 10.3390/jimaging6060052 34460598 PMC8321083

[B41] Smith S. M. Jenkinson M. Woolrich M. W. Beckmann C. F. Behrens T. E. Johansen-Berg H.. (2004). Advances in functional and structural mr image analysis and implementation as FSL. Neuroimage 23, S208–S219. 10.1016/j.neuroimage.2004.07.051 15501092

[B42] Souza R. Lucena O. Garrafa J. Gobbi D. Saluzzi M. Appenzeller S.. (2018). An open, multi-vendor, multi-field-strength brain MR dataset and analysis of publicly available skull stripping methods agreement. Neuroimage 170, 482–494. 10.1016/j.neuroimage.2017.08.021 28807870

[B43] Stanley E. Wilms M. Mouches P. Forkert N. (2023). “Exploring the role of explainability for uncovering bias in deep learning-based medical image analysis,” in Medical Imaging with Deep Learning, short paper track.

[B44] Stanley E. A. Wilms M. Mouches P. Forkert N. D. (2022). Fairness-related performance and explainability effects in deep learning models for brain image analysis. J. Med. Imaging 9:061102. 10.1117/1.JMI.9.6.061102 36046104 PMC9412191

[B45] Stites S. D. Cao H. James R. Harkins K. Coykendall C. Flatt J. D.. (2023). A systematic review of measures of gender and biological sex: exploring candidates for Alzheimer's disease and related dementias (AD/ADRD) research. Alzheimers Dement. 15:e12359. 10.1002/dad2.12359 36845632 PMC9943901

[B46] Taylor J. R. Williams N. Cusack R. Auer T. Shafto M. A. Dixon M.. (2017). The cambridge Centre for ageing and neuroscience (cam-can) data repository: structural and functional MRI, MEG, and cognitive data from a cross-sectional adult lifespan sample. Neuroimage 144, 262–269. 10.1016/j.neuroimage.2015.09.018 26375206 PMC5182075

[B47] Wiersch L. Hamdan S. Hoffstaedter F. Votinov M. Habel U. Clemens B.. (2023). Accurate sex prediction of cisgender and transgender individuals without brain size bias. Sci. Rep. 13:13868. 10.1038/s41598-023-37508-z 37620339 PMC10449927

[B48] Williams C. M. Peyre H. Toro R. Ramus F. (2021). Neuroanatomical norms in the UK biobank: the impact of allometric scaling, sex, and age. Hum. Brain Mapp. 42, 4623–4642. 10.1002/hbm.25572 34268815 PMC8410561

[B49] Wolberg A. Tripathy K. Kapoor N. (2024). Homonymous Hemianopsia. Treasure Island, FL: StatPearls Publishing. 32644355

[B50] Young J. E. Wu M. Hunsberger H. C. (2023). Editorial: Sex and gender differences in neurodegenerative diseases. Front. Neurosci. 17:1175674. 10.3389/fnins.2023.1175674 37008208 PMC10061136

[B51] Zhang Y. Qiu C. Lindberg O. Bronge L. Aspelin P. Bäckman L.. (2010). Acceleration of hippocampal atrophy in a non-demented elderly population: the snac-k study. Int. Psychogeriatr. 22, 14–25. 10.1017/S1041610209991396 19958567

[B52] Zhou S. K. Greenspan H. Davatzikos C. Duncan J. S. Van Ginneken B. Madabhushi A.. (2021). A review of deep learning in medical imaging: imaging traits, technology trends, case studies with progress highlights, and future promises. Proc. IEEE 109, 820–838. 10.1109/JPROC.2021.3054390 37786449 PMC10544772

