# Supplementary material for: Sex differences in brain MRI using deep learning toward fairer healthcare outcomes
Source: Front Comput Neurosci. 2024 Nov 13;18:1452457. doi: 10.3389/fncom.2024.1452457 (PMC11598355; doi:10.3389/fncom.2024.1452457)
Supplement: Supplementary file 1 [file Data_Sheet_1.PDF]

frontiers

Supplementary Material

1 SUPPLEMENTARY TABLES

Table S1: Unweighted and weighted saliency scores are presented for each brain region in the averaged sex differences saliency map for 98 correctly classified samples (50% female, 50% male) within a TIV range of 1378 to 1666 ml. These brain regions were identified from the CerebrA atlas.

Region Name	Saliency Score (%)	Weighted Saliency Score (%)
Left Entorhinal	98.85	98.85
Right Pars Orbitalis	91.76	89.29
Right Pars Triangularis	85.19	74.31
Right Pallidum	72.02	54.33
Right Basal Forebrain	87.50	49.02
Right Optic Chiasm	71.26	39.79
Left Rostral Anterior Cingulate	67.40	37.28
Right Pars Opercularis	57.70	31.01
Right Entorhinal	59.94	28.53
Right Lateral Orbitofrontal	52.87	21.86
Right Inferior temporal	46.92	20.09
Left Optic Chiasm	42.48	18.99
Right Accumbens Area	46.65	18.28
Right Lateral Occipital	41.65	14.71
Left Inferior temporal	32.53	12.38
Right Cerebellum Gray Matter	32.63	11.52
Left Amygdala	32.97	11.19
Right Insula	36.80	10.84
Left Superior Frontal	32.66	10.47
Left Parahippocampal	33.12	10.28
Right Putamen	29.72	10.26
Left Inferior Lateral Ventricle	31.35	9.75
Left Caudal Anterior Cingulate	30.28	9.37
Left Cerebellum White Matter	29.15	8.99
Left Pars Triangularis	27.57	8.27
Left Cerebellum Gray Matter	27.73	7.95
Right Superior Temporal	27.25	7.82
Right Rostral Anterior Cingulate	26.72	7.61
Left Medial Orbitofrontal	24.19	7.55
Left Brainstem	26.74	7.33
Right Brainstem	24.02	7.05

Table S1: (continued)

Region Name	Saliency Score (%)	Weighted Saliency Score (%)
Left Ventral Diencephalon	23.70	6.27
Left Hippocampus	20.81	5.83
Left Basal Forebrain	18.23	5.55
Right Rostral Middle Frontal	20.04	5.05
Left Superior Temporal	18.59	4.99
Right Caudate	20.82	4.92
Left Pericalcarine	20.50	4.86
Left Lateral Occipital	18.73	4.80
Left Paracentral	17.66	4.75
Right Cerebellum White Matter	16.22	4.11
Left Lateral Orbitofrontal	16.33	4.10
Right Middle Temporal	16.25	4.04
Right Thalamus	16.76	3.76
Right Medial Orbitofrontal	14.77	3.68
Right Superior Frontal	15.39	3.58
Left Fusiform	15.54	3.44
Left Pars Orbitalis	12.48	3.05
Left Middle Temporal	12.69	2.85
Left Third Ventricle	12.07	2.57
Right Amygdala	11.27	2.48
Right Ventral Diencephalon	11.66	2.40
Left Pars Opercularis	10.02	2.19
Left Transverse Temporal	10.84	2.13
Left Precuneus	8.89	1.61
Right Precentral	8.42	1.44
Right Fusiform	6.69	1.29
Right Caudal Anterior Cingulate	7.04	1.23
Left Lingual	6.96	1.21
Left Lateral Ventricle	8.76	1.17
Left Accumbens Area	5.97	1.14
Right Postcentral	5.34	0.88
Left Rostral Middle Frontal	4.42	0.87
Left Caudate	5.58	0.81
Left Thalamus	4.98	0.79
Right Lateral Ventricle	5.59	0.73
Left Cuneus	3.52	0.58
Left Pallidum	2.12	0.43
Left Isthmus Cingulate	3.19	0.42
Right Pericalcarine	2.13	0.38
Left Postcentral	2.41	0.36
Right Lingual	1.97	0.31
Right Inferior Parietal	1.94	0.31

Table S1: (continued)

Region Name	Saliency Score (%)	Weighted Saliency Score (%)
Right Superior Parietal	1.67	0.28
Right Inferior Lateral Ventricle	1.98	0.23
Right Hippocampus	1.73	0.20
Right Third Ventricle	1.18	0.16
Left Putamen	1.16	0.13
Left Precentral	0.68	0.11
Left Insula	0.87	0.10
Right Supramarginal	0.57	0.08
Right Precuneus	0.42	0.06
Left Fourth Ventricle	0.33	0.04
Right Paracentral	0.23	0.04
Right Vermal lobules VIII-X	0.19	0.03
Left Inferior Parietal	0.20	0.02
Right Fourth Ventricle	0.13	0.01
Left Superior Parietal	0.07	0.01
Left Posterior Cingulate	0.02	0.00
Left Caudal Middle Frontal	0.01	0.00
Right Caudal Middle Frontal	0.00	0.00
Left Vermal lobules VI-VII	0.00	0.00
Left Vermal lobules I-V	0.00	0.00
Right Vermal lobules VI-VII	0.00	0.00
Right Transverse Temporal	0.00	0.00
Right Vermal lobules I-V	0.00	0.00
Left Vermal lobules VIII-X	0.00	0.00
Right Cuneus	0.00	0.00
Right Parahippocampal	0.00	0.00
Right Posterior Cingulate	0.00	0.00
Right Isthmus Cingulate	0.00	0.00
Left Supramarginal	0.00	0.00
